#  Effects of Cabergoline administration on uterine perfusion in women with polycystic ovary syndrome

**DOI:** 10.12669/pjms.294.3558

**Published:** 2013

**Authors:** Robabeh Mohammadbygi, Sayedeh Reyhaneh Yousefi, Sholeh Shahghaybi, Shokrollah Zandi, Karim Sharifi, Fardin Gharibi

**Affiliations:** 1 Robabeh Mohammadbygi, Associate Professor of Obstetrics and Gynecology, Kurdistan University of Medical Sciences, Sanandaj, Iran.; 2 Sayedeh Reyhaneh Yousefi, Resident Physician of Obstetrics and Gynecology, Kurdistan University of Medical Sciences, Sanandaj, Iran.; 3 Sholeh Shahghaybi, Associate Professor of Obstetrics and Gynecology, Kurdistan University of Medical Sciences, Sanandaj, Iran.; 4 Shokrollah Zandi, Resident physician of Neurosurgery, MPH Shahid Beheshti University of Medical Sciences, Iran.; 5 Karim Sharifi, Assistant Professor of Radiology, Kurdistan University of Medical Sciences, Sanandaj, Iran.; 6 Fardin Gharibi, MsPH Health Management, Deputy of research, Kurdistan University of Medical Sciences, Sanandaj, Iran.

**Keywords:** Polycystic ovarian syndrome, Cabergoline, Uterine artery resistance, Resistance Index, Pulsatility Index

## Abstract

***Objectives:*** The aim of this study was to determine the effects of Cabergoline administration on uterine blood flow in women suffering from polycystic ovary syndrome (PCOS).

***Methods:*** This study is a randomized, controlled, triple-blind trial which is tested on 40 women who were randomly divided into two groups of 20 people and using a *randomized block* design during which the subjects were assessed and included. They were suffering from polycystic ovarian syndrome. Inclusion criteria were classically defined PCOS criteria including: oligomenorrhea or amenorrhea, clinical or Laboratory findings based on increase in blood level androgen (testosterone) and ultrasound confirmation of PCOS. Exclusion criteria were Pregnancy, lactation, *Dopamine Agonist Therapy*. After selection of intervention and placebo groups, primary control Doppler ultrasound was done for both groups. Then a weekly dose of Cabergoline 0.5 mg was administered to intervention group for duration of 12 weeks. Placebo group were administered placebo in the same fashion. At the end of 12 weeks, Doppler ultrasound was performed and the results were recorded in the check lists.

***Results:*** No significant difference was noticed in both groups with respect to their age, employment, level of education, type of infertility, duration of marriage, and results of RI and PI before intervention. Later PCOS patients under the treatment of Cabergoline showed a significant increase in uterine blood flow Pulsatility Index (PI) before 2.65±0.52 and after 1.98±0.52 and RI before 0.85 and after intervention 0.77), yet no significant difference were found in PCOS patient under the treatment of placebo.

***Conclusion:*** PCOS patients were shown to have more resistance in uterine blood flow than healthy people; however, Cabergoline administration proved to increase uterine blood perfusion and regulate menstruation cycle.

## INTRODUCTION

Major features of polycystic ovarian syndrome (PCOS), a common disorder affecting women of childbearing age, include oligomenorrhea or amenorrhea with clinical or laboratory signs of hyperandrogenemia. PCOS is probably an oligogenic or polygenic disorder.^1^ Its signs and symptoms starts in puberty.^2^ PCOS is the most common cause of female infertility. Many women with polycystic ovary syndrome experience infrequent ovulation or lack of ovulation altogether and may face many challenges to become pregnant.^1^^,^^3^

Chronic lack of ovulation increases any woman's risk of endometrial (uterine) or breast cancer. To avoid these undesirable outcomes, physicians should diagnose and decide to treat the disorder. PCOS occurs in almost 4-6 % of fertile women.^4^^,^^5^

In line with Rotterdam public meeting, American Society for Reproductive Medicine and European Society of Human Reproduction and Embryology (in 2003), diagnosis of PCOS is made based on the following criteria:

- Oligo-ovulation or anovulation manifested by oligomenorrhea or amenorrhea.
- Biochemical evidence of blood androgen excess.
- Polycystic ovaries as defined on ultrasonography.^1^^,^^2^

Different studies using Doppler ultrasound shows higher resistance in uterine blood flow of these cases.^5^^-^^8^ As suggested by several authors, Doppler evaluation of ovarian and uterine arteries, are additional ways to confirm the diagnosis. Knowing the great role of uterine blood flow in endometrial receptivity, increase in pulsatility index (PI) of uterine arteries have been reported in infertile couples who had little chances of pregnancy.^9^

Higher resistance in uterine blood flow of PCO patients may reduce endometrial receptivity and eventually, increased chances of miscarriage,^10^ as well as having a very negative effect on the outcome of pregnancy.^11^ Some studies have revealed the effects of medications in reducing uterine blood flow resistance in PCOS patients effect of Cabergoline on uterine blood flow of women suffering from PCOS shows that it can increase the uterine blood flow.^12^^,^^13^

Cabergoline, ergot-derived dopamine agonists with a very long half life, is an effective prolactine suppressor. Cabergoline oral administration contains a weekly dose of 0.5 – 3 mg, which could be increased, if needed, to twice a week. This medicine has slight dopamine agonistic side effects, headache being the most common one. Treatment in the very beginning should start with a partial dose (half a pill) at bedtime with a small amount of food. Low incidence of side effects and its weekly dose has made Cabergoline a choice drug for treatment of related diseases**.**^1^

There is high prevalence of PCOS and greater infertility in women who suffer from the disease. In addition there is uterine blood flow resistance on endometrial receptivity. Small number of studies have been conducted on effect of Cabergoline on the uterine blood flow and there is lack of such studies in our country. All this encouraged us to conduct this study with the aim of identifying effects of Cabergoline on uterine blood flow in women suffering from PCOS.

## METHODS

The study design was randomized, controlled, triple-blind trial. Population under study was fertile women suffering from PCOS, who were living in Sanandaj. Inclusion criteria were classically defined PCOS women, 20 years of age and more. Exclusion criteria were Pregnancy, lactation, side effects of Cabergoline consumption including sickness, vomiting, vision defect, and headache.

Study sample comprised of 40 women suffering from PCOS who were randomly divided into two groups of 20 people Using a *randomized block* design in which the subjects were assessed and put in *blocks* of *four*. From the placebo group one sample was excluded from the study due to pregnancy. Informed consent was obtained from the participants. Patients over 20 years with PCOS were chosen among selected primary studied cases that referred to the Besat gynecology clinic. After explaining the research plan and obtaining their consent, they were randomly assigned into control and placebo groups. Color Doppler ultrasound was performed from the ascending main branch of the uterine artery on both groups. Although both sides (right and left) were evaluated, however our focus was on the right side. In order to avoid circadian rhythm influence, all ultrasounds were taken from 5 to 8 o’clock in the evening and every Doppler ultrasound was performed by the same workforce and device.

Ultrasound was done during the early follicular phase of menstrual cycle (days 2 – 6). In the second phase of the study Italian made oral Cabergoline was administered after dinner to intervention group with a dose of 0.5 mg per week for 12 weeks. Then placebo was administered after dinner, once in a week for 12 weeks, to the control group. During the third month after treatment, Doppler ultrasound evaluation was performed on the ascending main branch of the uterine artery on the early follicular phase and the results were recorded using a checklist. After data entry, data were analyzed using SPSS 18. showed that data were non-normally distributed. Descriptive and analytical statistics (Mann Whitney U) test was used to compare the uterine blood flow in two groups and Wilkakson test was used to compare before and after uterine blood flow in groups.

## RESULTS

There were no significant difference between the mean difference of demographic data including: Age, Occupation, Education, Type of infertility, Duration of marriage, and duration of infertility.)Table-I)

**Table-I:** *Distributions* and *frequency* of demographic characteristics in the two groups

*Group* *Variable*	*Gabergoline*	*Placebo*	*P*
	*Number (%)*	*Number (%)*	
*Occupation: * House keeper Employed	16(80) 4(20)	14(73.7) 5(26.3)	*0.86
*Education: * Under secondary School Under graduate Graduate	7(35) 7(35) 6(30)	8(42.1) 7(36.8) 4(21.1)	*0.85
*Type of infertility: * Primary Secondary	11(84.6) 2(15.4)	11(78.6) 3(21.4)	*0.98
Age (Mean ± SD)	25±4.5	24.5±4.6	**0.73
Duration of marriage	5.9±5.5	4.9±4.0	**0.72
Duration of infertility	4.9±3.9	3.9±3.5	**0.52

*Fisher's Exact Test          **Mann-Whitney U Test

**Table-II:** Comparison of PI and RI in Cabergoline group before and after the intervention

*Variable*	*Group*	*Number*	*Mean*	*SD*	*P*
*PI before	Cabergoline	20	2.65	0.5	0.09
	placebo	19	2.37	0.5	
**RI before	Cabergoline	20	0.85	0.07	0.49
	placebo	19	0.84	0.07	
PI after	Cabergoline	20	1.98	0.5	0.03
	placebo	19	2.38	0.5	
RI after	Cabergoline	20	0.77	0.1	0.015
	placebo	19	0.85	0.06	

*pulsatility index ** Uterine artery resistance

**Table-III:** Comparison of PI and RI, before and after intervention in placebo group

*Group*			*Mean*	*SD*	*P*
Placebo	RI	Before	0.84	0.07	0.81
		After	0.85	0.06	
	PI	Before	2.37	0.50	0.95
		after	2.38	0.49	
Cabergoline	RI	Before	0.85	0.07	0.01
		After	0.77	0.1	
	PI	Before	2.65	0.52	0.001
		after	1.98	0.52	

Before intervention, PI was 2.65±0.5 in Cabergoline group and 2.37±0.5 in placebo group, did not show a significant difference between the two groups (p=0.09). RI were 0.85±0.07 in Cabergoline group and 0.84±0.07 in placebo group, which also did not show a significant difference between the two groups (p=0.49). After intervention, PI was (1.98±0.5) in Cabergoline group and (2.38±0.5) in placebo group, therefore the difference between those two groups is said to be statistically significant (p=0.03).

After intervention, RI was 0.77±0.1 in medication group and 0.85±0.06 in placebo group; therefore the difference between those two groups is said to be statistically significant (p=0.015). (Table-II).

In placebo group, before intervention the average Uterine artery resistance (RI) was 0.84±0.07 and after intervention it was 0.85±0.06, therefore no significant relation between two groups was found (p=0.81). Furthermore the average of PI was 2.37±0.5 before intervention and 2.38±0.5 after intervention, therefore no significant relation between two groups was found after intervention (p=0.953)

In Cabergoline group, the average of RI was 0.85±0.07 before intervention, coming down to 0.77±0.1 after intervention, therefore the difference was statistically significant (p=0.01). The average of PI was 2.65±0.5 before intervention and 1.98±0.5 after intervention, therefore the difference was statistically significant (p=0.001). (Table-III).

 Before treatment, every patient in the study had oligomenorrhea, yet after treatment 19 women showed regular menstrual cycle. Fisher's exact test 0.01, showed a significant difference between intervention and control group regarding regular menstrual cycle (p= 0.86), of which 70% was due to Cabergoline administration (73.3% compared with 26.3% in control group). No other side effects occurred in the patients; however, one of the subjects was removed from the study due to pregnancy.

## DISCUSSION

Previous studies have shown that, women with increased uterine artery *pulsatility index* (PI) has less chance of pregnancy.^14^ Pathologic uterine blood flow in PCOS cases caused reduced endometrial receptivity, therefore increased chances of abortion. Accordingly, in PCOS patients, role of certain medication is more important. One of these medications is Cabergoline and its efficacy has been studied worldwide.^13^

Our study, with the focus on uterine artery resistance, was carried on 40 patients with diagnosed PCOS in Gynecology clinic at Besat Hospital. Obviously one of the cases was removed from the study owing to pregnancy. In this study no statistically significant difference were seen between intervention and control groups based on age, occupation, level of education, duration of marriage, duration of infertility, type of infertility and regular menstrual cycle. 

In patients under study, the average of PI with (2.51±0.52) was less than PI average comparing to other similar studies (3.98±0.52 and 2.97±0.9). This could be explained by racial and environmental differences; hence further national studies are required to achieve more general results.^10^^,^^12^

In the later stage of our study, average of PI in our PCOS patients in the intervention group decreased significantly to 1.98±052 compared with 2.65±0.52 before treatment with Cabergoline. (p<0.001).In a similar study, average of PI in the control group who were healthy, was estimated to be (1.59±0.2).^12^ Therefore no significant differences was found in the average of PI in PCOS patients before and after placebo administration (2.37±0.5 2.38±0.49) (P<0.46). In PCO patient who where under treatment by Cabergoline, RI was 0.85 before intervention, with standard deviation of 0.07, which reduced to 0.77 after intervention, with standard deviation of 0.1, consequently the difference was statistically significant (p<0.01). Uterine artery resistance (RI) was 0.84, with standard deviation of 0.07, in the group under placebo treatment and 0.85, with standard deviation of 0.06, in the group after placebo treatment, however the difference was not statistically significant. (p<0.8)

Our study which is one of the few studies showing the impact of Cabergoline on uterine artery blood flow, was consistent with previous other universal studies; however, PCOS patients in our study showed to have more uterine artery resistance.^5^^-^^8^^,^^14^^-^^16^

According to findings from this study, Cabergoline can increase uterine blood flow, which could be attributed to the hormonal variation in the patients involved. Similar other studies have been done on the impact of cabergoine on ovarian and adrenal androgen as well as a positive relation between uterine artery PI and serum androgen.^8^^,^^12^ These studies suggest that androgen decreases markedly during Cabergoline treatment hence decrease in the blood level of androgen could explain return of menstrual cycle and improved uterine blood flow. Participants in the study did not have hyperprolactinemia or galactorrhea. These findings could be a new beginning in the treatment of patients with PCOS.

Cabergoline could be used as an alternative treatment in these patients, knowing that this drug is well tolerated and has no side effects. This medicine could be prescribed to young patients who are suffering from PCOS and are determined to get pregnant. Due to effects of Cabergoline on menstruation regularity and improvement in uterine perfusion, treatment with Cabergoline can facilitate pregnancy in PCOS patients. Cabergoline have no side effects such as ovarian hyperstimulation syndrome (OHSS) or multiple pregnancies which could be seen in other ovarian stimulation methods; therefore usage of Cabergoline could be considered as safe for pregnancies.
